# Handgrip Strength-Related Factors in a Colombian Hypertensive Population: A Cross-Sectional Study

**DOI:** 10.3390/ijerph19063726

**Published:** 2022-03-21

**Authors:** Yulieth Rivas-Campo, Elsa Patricia Muñoz-Laverde, Agustín Aibar-Almazán, José Daniel Jiménez-García, Antonio Martínez-Amat, Patricia Alexandra García-Garro, Juan Miguel Muñoz-Perete, Manuel Garcia-Sillero, Yolanda Castellote-Caballero

**Affiliations:** 1 Faculty of Human and Social Sciences, University of San Buenaventura, Cali, Santiago de Cali 760031, Colombia; yrivasc@usbcali.edu.co; 2 School of Public Health, Faculty of Health, University of Valle, Santiago de Cali 760043, Colombia; elsa.munoz@correounivalle.edu.co; 3 Department of Health Sciences, Faculty of Health Sciences, University of Jaén, 23071 Jaén, Spain; josedanieljimenezgarcia@gmail.com (J.D.J.-G.); amamat@ujaen.es (A.M.-A.); palexandragarcia@admon.uniajc.edu.co (P.A.G.-G.); jmmunoz@ujaen.es (J.M.M.-P.); mycastel@ujaen.es (Y.C.-C.); 4 Faculty of Sport Sciences, EADE-University of Wales Trinity Saint David, 29018 Málaga, Spain; manuelgarcia@eade.es; 5 Laboratory Fivestars, 29018 Málaga, Spain

**Keywords:** handgrip strength, high blood pressure, dynamometry, functional evaluation, physical activity

## Abstract

(1) Background: This study determined the factors associated with manual grip strength in people with high blood pressure (HBP); (2) Methods: 219 subjects participated in this cross-sectional study, which evaluated muscle strength (manual dynamometer), sociodemographic factors, clinical characteristics, level of physical activity (International Physical Activity Questionnaire-IPAQ score), and depression (Zung’s Depression Self-Rating Scale); (3) Results: The bivariate analysis found that handgrip strength in people with HPB was associated with sex (*p* = 0.000), age (*p* = 0.000), ethnicity (*p* = 0.019), smoking habits (*p* = 0.037), alcohol consumption (*p* = 0.004), diastolic blood pressure (*p* = 0.012), weight (*p* = 0.000), height (*p* = 0.000), measurement of waist circumference (*p* = 0.002), depression (*p* = 0.041), and IPAQ score (*p* = 0.000). Regardless of being male or female, handgrip strength was associated with age (*p* = 0.009), IPAQ (*p* = 0.000), weight (*p* = 0.038), height (*p* = 0.000), DPB units (*p* = 0.043), and depression (*p* = 0.020). The multivariate generalized linear gamma regression model showed that the coefficient with the greatest weight, regardless of sex, was age (*p* = 0.043), level of physical activity (24% more at high level than at low level, *p* = 0.031), and depression (moderate/severe depression level) associated with lower handgrip strength (*p* = 0.025); (4) Conclusions: Handgrip strength showed an association with level of physical activity, age, and level of depression in a middle-aged population with HBP.

## 1. Introduction

Chronic non-communicable diseases (CNCD) account for approximately 60% of all mortality worldwide [1], and among them cardiovascular diseases are some of the most common causes of disability and premature death (defined as that occurring to individuals aged 30 to 70) in the world at large as well as in the specific region under study [2]. More specifically, HBP is the main risk factor for premature death due to a cardiovascular event and appears as the second most common cause for disability worldwide [3]. Likewise, HBP was identified as one of the main concerns in the Global Burden of Disease report of 2010, in which it ranked fifth among causes of general mortality at a rate of 17.2 per 100,000 inhabitants [4]. That report included calculations that high blood pressure caused the loss of 170 million years of life in 2013 [5,6].

The prevalence of high blood pressure in Colombia is 28%. Among individuals 18 to 69 years old who visited the Colombian General System of Social Security in Health in 2015, 7.2% did so because of high blood pressure levels [7]. Specifically, in Cali, HBP ranks second among the fifteen most common causes of mortality, which makes it a key target for monitoring programs in healthcare institutions [8].

In recently years, HBP has been found to cause a decrease in physical function [5], higher disability levels [9], and several comorbidities which add to the loss of functional ability [10]. Due to potential complications of HBP, there is widespread concern to measure the extent to which this disease affects physical function [11,12].

Handgrip strength levels help determine the risk for functional limitation [13] and are an indicator of health conditions and functional ability with direct associations with morbidity and mortality [4,12]. The handgrip strength levels predict the risk of mortality by any causes [6,12], and when high is inversely associated with heart disease and cerebrovascular accidents [14,15], metabolic risk [16], and mental health [17]. Despite these associations, handgrip strength is rarely used as a marker of disease risk in primary care settings [18], although these measurements may be useful for health education action planning. Research to date has proven the association between HBP and handgrip strength, showing that individuals suffering from HBP experience accelerated functional loss. This is first apparent as a decrease in the ability to perform strength-related tasks, and later evolves towards disability [19,20]. However, these conclusions are derived from studies on populations over 60, when HBP appears at much younger ages [21].

Furthermore, a growing interest in the study of the association of handgrip strength with sociodemographics and lifestyle has begun to emerge [22], and adolescents with a higher socioeconomic status or who do not comply with physical activity recommendations are at greater risk of having a lower level of handgrip strength [23]. However, despite the evidence suggesting this influence, few studies have investigated the factors associated with handgrip strength, and none has studied these factors in people with HBP. Considering the above, the aim of the present study was to determine the factors associated with handgrip strength among a population 35 to 64 years old, with a diagnosis of HBP, who attended a monitoring program at a healthcare facility in Santiago de Cali. Thus, sociodemographic variables such as occupation, ethnicity, and economic stratum are expected to influence prehensile strength values. Regarding clinical variables, conditions such as overweight, abdominal obesity and high levels of depression are expected to be associated with lower prehensile strength measurements.

## 2. Materials and Methods

### 2.1. Study Design and Participants

An analytical cross-sectional study was carried out in January and February 2020 for which 3720 individuals between 35 and 64 years of age were initially contacted. The selection of subjects was carried out through the registry of the HBP program of the healthcare facility in Cali (Colombia). The people were contacted when leaving a control appointment of the program, the objectives were explained to them of the research, and those who agreed to participate signed a written informed consent form and an appointment was made for the data collection process and the assessment of prehensile strength. The study was approved by the Institutional Ethics Committee (CD 037688-S010010105) and the Health School Ethics Committee (008-19).

**Figure 1:**
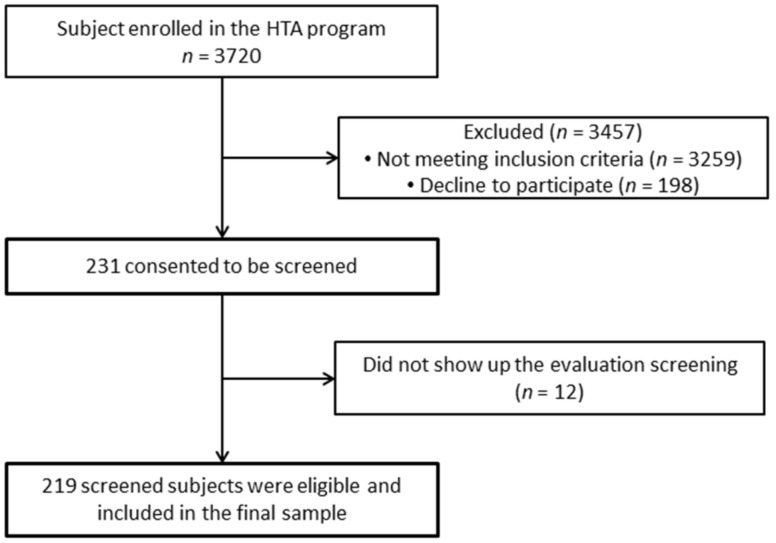
Flowchart diagram of the participants in this study.

Men and women of ages 35 to 64 were included who had been diagnosed with HBP (140/90 mmHg) for at least six months, attended the chronic disease program, and who accepted to take part in the study after being informed of its purpose and procedure. Participants were excluded who had been diagnosed with cancer, diabetes, pulmonary hypertension, kidney failure, heart failure, psychiatric disorders, or any neurological or cognitive alteration, as well as those with an infection, including human immunodeficiency virus (HIV). Persons with medical records of musculoskeletal diseases diagnosed in the upper limb (either general or occupational disease) were excluded, as well as injuries with less than 2 years of evolution (general, traffic accident, or occupational). For those who at the time of the assessment referred pain or other symptoms that led to suspect any pathology but who had not yet received a medical diagnosis, the physiotherapist performed semiology tests and decided on their participation in the study. Figure 1 shows a flowchart diagram of the participants.

### 2.2. Study Variables

#### 2.2.1. Sociodemographic Characteristics

Sociodemographic data such as educational level, occupation, marital status, ethnicity, and socioeconomic status were taken from a survey administered by the researcher and by the qualified professional, which was applied in an appointment arranged with the participant in the assigned clinic by the Institution for research.

#### 2.2.2. Clinical Characteristics

The records of the healthcare institution provided data concerning time since HBP diagnosis, systolic (SBP) and diastolic (DBP) blood pressure values, and use of medication. An additional survey administered by the same interviewers collected data on smoking habits, alcohol consumption, physical activity, and depression.

The data of the variables weight, height, and abdominal perimeter were obtained by measurement by the researcher.

Weight was recorded using a mechanical scale (Kenwel Dt612^®^ Omagh, North Ireland); height was measured using a height rod fixed to the wall; and abdominal circumference was measured using a 2-m measuring tape with a 1-mm allowance and following the measuring protocols of the International Society for the Advancement of Kinanthropometry [24].

Zung’s abbreviated self-rating depression scale was used to determine levels of depression among adults living in the community [25]. Its Colombian version was validated by Campo et al. [26].

The International Physical Activity Questionnaire (IPAQ) was used to establish individual levels of physical activity. Its Spanish-language Colombian version was validated in 2003 [27].

The duration of the assessment was approximately 40 min on average (±5 min) per participant. Initially, the 20-min sociodemographic survey was applied, followed by the IPAQ and Zung scales, which were self-administered.

For taking body weight, the evaluator had to make sure that the scale was at zero; the participant stood without shoes in the center of the plate, distributing the weight equally on both supports, arms parallel to the body without holding them during measurement, with an upright trunk, and looking straight ahead. To measure height, the stadiometer was adapted to the wall, positioning the participant in an upright position, without shoes, with the upper limbs on both sides of the body, the palms and fingers of the hands relaxed downwards while standing, with the weight distributed equally on both feet. The evaluator confirmed that the feet did not leave the ground, and that the Frankfurt plane was maintained (the imaginary line drawn from the lower end of the orbit to the upper edge of the external auditory canal is aligned horizontally); the table was supported firmly on the Vertex (Boston, MA, USA), and the hair on the top-most portion of the head was flattened as much as possible. The measurement was taken at the end of a deep expiration determining the linear vertical distance from the Vertex to the intra heel-floor line [24].

From these previous data (weight and height), the body mass index (BMI) was calculated by applying a formula that divides the weight, represented in kg, by the value of the height squared, represented in meters squared. That value was recorded in the format.

For waist circumference, the subject stood with arms crossed across the chest; it was located at the smallest perimeter of the abdomen between the costal margin and the iliac crest at the end of expiration, taking the reading in front of or slightly lateral to the subject [24].

#### 2.2.3. Muscle Strength

Muscle strength was measured using a grip dynamometer (TKK5001 Grip-A, Takei, Japan) according to the Jamar dynamometer protocol. The participant was placed in a standing position with a straight back, the shoulder adducted and in neutral rotation, the elbow flexed at 90°, the forearm in a neutral position, and the wrist in a neutral position. The participant was asked to squeeze a device that measures force (dynamometer), which is adapted to the length of the hand so that it forms an angle of 90° between the first and second phalanges of each finger. The force executed in both the right and left hands was be taken. The measurement were carried out repeatedly on three occasions with an interval of five minutes between each of the measurements, which followed the evaluation protocol [28].

### 2.3. Sample Size Calculation

Sample size was calculated by means of EPIDAT 4.1 software (Epidemiology Service (Atlanta, GA, USA; Junta de Galicia, Santiago de Compostela, Spain); Pan American (Key West, FL, USA); World Health Organization: PAHO-WHO (Washington, DC, USA); and Universidad CES of Colombia (Medellín, Colombia). For our study, the following parameters were applied: finite total population of 3720, 3% precision, 95% reliability, 18.85% expected standard deviation according to the findings of Triana et al. [29], and 30% non-response. The resulting calculation for a random sampling design was 146 participants. After adjusting for design effect (1.5), the final sample size was 219.

### 2.4. Statistical Analysis

The database was processed in Stata 14 (USA). The Kolmogorov–Smirnov test was applied for checking normality. In the univariate analysis, quantitative sociodemographic and clinical variables with a normal distribution were presented as means and standard deviation values (SD), and variables with a non-normal distribution using their median and interquartile range (IQR). Categorical variables with low frequency were recategorized. For the bivariate analysis, the median of the handgrip strength value was obtained for each group of the clinical and sociodemographic factors. These values were compared; dichotomous variables were analyzed through the Wilcoxon–Mann–Whitney test, and polychotomous exposition variables with the Kruskal–Wallis test.

A simple model regression analysis was performed on each variable to determine the coefficient of the gamma generalized linear model. For all hypothesis contrast tests, a significance level of 5% (*p* = 0.05) was used. To identify potential confounding or effect-modifying factors, stratification was applied according to sex, age, and time since HBP was diagnosed. Variables displaying statistical significance were integrated in the multiple (gamma) generalized regression model. The adjusted regression coefficient was calculated, and the most suitable model was tested using the backward method.

## 3. Results

A total of 219 men and women took part in the study, with a median age of 57 (IQR 11, and a confidence interval (CI) of 95% 56–59). Most of the participants were women (67.6%), had a secondary education attainment level (57.1%), and were married (44.3%). As for the clinical variables (Table 1), the median value for the time since diagnosis of HBP in the observed group was 7 years (IQR 6 with CI 95% 6–8), and 79.9% had systolic pressure values over 120 mmHg (IQR 10 with CI 95% 120–124.52). Additionally, 60.7% displayed diastolic pressure values above 80 mmHg. Regarding waist circumference, a median of 93.5 cm (IQR 18) was calculated, with 85.38% displaying values that placed them at risk of heart disease (Table 1).

A total of 92.2% of participants was under medication for blood pressure control. It was reported that 92.2% of the population did not smoke or drink alcohol (81.3%). According to the IPAQ scale, 64.4% of the population was deemed to engage in low levels of physical activity. The Zung scale showed that 48.4% exhibited signs of mild depression, 21.5% moderate, and 0.5% severe depression. The handgrip strength (non-normal distribution), with a median value in the right hand of 24.67 kgF (IQR 15) and left hand with a median value of 23.66 kgF (IQR 14). The comparison of median values among related groups determined that there were no statistical differences among both measurements (*p* = 0.080), and no handgrip strength differences due to hand dominance (*p* = 0.900). For this reason, the highest value (of the attempts) of the right hand was taken.

The Wilcoxon–Mann–Whitney test found statistically significant differences in handgrip strength according to sex (*p* = 0.000), ethnicity (*p* = 0.019), smoking habits (*p* = 0.017), alcohol consumption (*p* = 0.016), and abdominal obesity (for waist circumference values amounting to heart disease risk) (*p* = 0.006). The Kruskal–Wallis test revealed statistically significant differences in handgrip strength median values by educational attainment (*p* = 0.038), occupation (*p* = 0.000), IPAQ rating (*p* = 0.000), and depression (*p* = 0.041) (Table 2).

The simple model (generalized gamma linear regression model) revealed the handgrip strength in a HBP population of age 35 to 64 can be associated with sex, showing that men have 1.76 units (kgF) higher the value of handgrip strength compared to women (*p* = 0.000). Age showed an inverse association (*p* = 0.001), indicating that when the age of the participants is 34 years old, the average handgrip strength is 34.55 kgF and for each year that age increases, the average is 0.98 times the reference value.

According to the ethnic group, it was estimated that the handgrip strength in people who self-recognized as black/mulatto/Afro-Colombian is on average 31.25 kgF and being of another ethnic group, the handgrip strength is 0.84 times the average of the black ethnic group (*p =* 0.026)

In the participants who were classified as low physical activity level according to the IPAQ, the average estimated handgrip strength was 24.82 kgF, and in those who were classified as level high, it was 43% higher than those classified as low level (*p* = 0.000).

For the classification of depression with Zung scale, an association with depression was found, showing that the average handgrip strength in persons with moderate/severe depression was 0.776 times compared to those without depression (*p* = 0.032).

Regarding the DPB, when it presented values of 80 mmHg, the average handgrip strength was 26.53 kgF, and with each millimeter of mercury that increased the DPB, the average handgrip strength decreased 0.99 units from the reference value (*p =* 0.012). Conversely, greater levels of handgrip strength were found with increasing height and weight (*p* = 0.000) (Table 3).

To evaluate confusion factors, each model was adjusted for sex and showed that regardless of being male or female, handgrip strength was associated with age (*p* = 0.009), IPAQ (*p* = 0.000), weight (*p* = 0.038), height (*p* = 0.000), and DPB units (*p* = 0.043) and depression (*p* = 0.020). No confounding factors were detected after adjusting for time since diagnosis, physical activity, or age rank.

A multivariate analysis, based on a gamma generalized linear model, showed that after adjusting for all the variables considered, the average handgrip strength in this HBP population aged 35 to 64 years was 23.94 kgF, and it showed an association with age, height, IPAQ level, Zung scale, and being single (Table 4). In search of the most parsimonious model, the backward method showed that the coefficients with the highest weights regardless of sex were age (*p* = 0.043), depression (*p* = 0.025), and IPAQ (*p* = 0.031) (Table 4). Goodness-of-fit evaluation was performed using the AIC (Akaike Information Criterion) and BIC (Bayesian Information Criterion) indices. The final model had an AIC of 1869.01 and a BIC of 1875.7 with respect to the model composed of all the variables that showed statistical significance in the bivariate (AIC of 1878.7 and a BIC of 1905.8), which supports that the model with fewer variables is the one with the best fit.

## 4. Discussion

The aim of this study was to establish the factors associated with handgrip strength in middle-aged hypertensive men and women. The main contributions of this study confirmed that after adjusting for sex in a multivariate model, handgrip strength showed association with age, height, IPAQ level, Zung scale, and singleness (marital status), but the most important variables (selected by the backward method) explaining the model were age (*p* = 0.043), depression (*p* = 0.025), and IPAQ (*p* = 0.031). Exploring these factors provides tools for the implementation of comprehensive health education interventions in different contexts, aimed at better understanding the health risks associated with low muscle strength levels [23] in people with HBP; it also helps to encourage prehensile strength measurements in clinical practice for healthcare providers and their patients [18].

Handgrip strength may be influenced by such factors as age, sex, and loss of muscle mass, which is why studies dealing with the matter have usually centered their focus on populations of older people [30,31]. However, there is sufficient evidence to support the notion that at other stages in the life cycle, one of the factors most closely linked to loss of handgrip strength is high blood pressure (*p* < 0.05) [19], with HBP individuals exhibiting significantly lower values of handgrip strength than those without the condition (63.5 vs. 71.5, *p* = 0.008) [20]. Additionally, handgrip strength has been found to be independently associated with some cardiovascular risk factors, such as obesity, hypertriglyceridemia, and high systolic blood pressure [27,28,32]. However, to date no studies have reported handgrip strength alterations associated with HBP-related factors.

Our results allowed us to establish the median handgrip strength for men (38.33 kgF), IQR 8 and women (21.58 kgF) IQR3 for each age in a HBP population, with reference values below those in Schlüssel’s reference table [33]. As for sociodemographic factors associated with handgrip strength, an inverse relation was verified to be in agreement with the muscle loss commonly experienced by individuals 30 years of age and over [34]. The average handgrip strength decreases 1% with each year of age that increases (*p* = 0.009).

When reviewing the literature, it can be established that factors such as marital status and educational level had not been considered in the exploration of the relationship of handgrip strength with sociodemographic factors in the HPB population. Regarding marital status, married participants were estimated to have an average of 26.73 kgF. In participants who reported being widowed, their average handgrip strength was 0.80 times the average of married people (*p =* 0.029), and in those who were separated, the average was 0.81 times the average of the handgrip strength of the reference category *p* = 0.085. In those who reported being widowed, no statistically significant association was found.

This study shows that schooling and socioeconomic status lose significance when analyzed independently of sex. This could probably be explained because handgrip strength represents a physical quality that reflects functionality and has been mainly related to biological factors, physical conditions, or activities that allow it to be trained.

For its part, the relationship found in the bivariate analysis in this research for occupation (the average prehensile force is 17% lower in those who are retired (*p* = 0.007) and 33% lower in people who perform household chores compared to the average of the prehensile force of those who work (*p* = 0.039)) had not been explored in preliminary studies.

Concerning the clinical variables, previous studies have reported that handgrip strength is approximately 10% stronger in the dominant hand than the non-dominant [35,36]. However, the differences observed in our research did not reach statistical significance (*p* = 0.900).

Evidence shows that handgrip strength is higher in individuals who scored lower on cardiovascular risk factors such as waist circumference [32]. Similarly, it has been established that high blood pressure is associated with a decrease in handgrip strength (average 9.32 kgF) compared to normotensive populations [20].

The results of this study indicate that handgrip strength in an HBP population is influenced by abdominal obesity, showing that in people with cardiovascular risk due to abdominal obesity, the average handgrip strength is 0.88 times the average of those without (*p =* 0.021 95% CI: 0.79–0.98). This is to be expected since the indicator “waist circumference” has been proposed as a good proxy for visceral adiposity in a wide age range [37]; the percentage of visceral fat has been significantly associated with hypertension in several populations [38,39,40]. Despite the above, according to the multivariate analysis, in the final model obtained by looking for the principle of parsimony, this variable was not associated with a statistically significant degree to variations in handgrip strength after adjusting for sex (*p* = 0.950), nor was it associated with time since diagnosis (*p =* 0.751), nor with the consumption of medications. These facts have never been reported in the literature.

The IPAQ questionnaire revealed a resulting score that is directly associated with handgrip strength; the simple model with bivariate analysis showed that with participants classified in the low level of physical activity, the average estimated handgrip strength was 24.82 kgF; in those with a medium level of physical activity, the average prehensile strength was 1.17 kgF units higher than the average of those in the low level *(p =* 0.007), and in those classified as high level, the average handgrip strength was estimated to be 1.43 kgF higher than the average of those in the low level (43% higher *p =* 0.000). This could be explained by the fact that strength is cultivated by engaging in frequent physical activity, and the questionnaire is very specific in asking about the time employed in a series of concrete activities over the last seven days.

In agreement with results found in the literature, anthropometric factors such as height and weight were found to be linked to handgrip strength; with the present study it was possible to estimate that when the average weight of the participants with HPB is 75 kg, the average handgrip strength is 26.78 kgF, and with each kilogram that increases, the average handgrip strength increases 1.01 kgF units (*p* = 0.000). Similarly, when the average height of the population is 161 cm, the average handgrip strength is 26.11 kgF, and with each cm that increases, the force increases 1.02 kgF (*p* = 0.000).

Contrastingly, BMI failed to exhibit an association (*p* = 0.910). In that regard, while some previous studies have reported such a link, others have found that association to be below the threshold of statistical significance [41].

One of the main factors associated with handgrip strength was sex, showing that men of all ages registered higher values. This could be explained by the higher proportion of muscle fibers in men and the fact that women can be influenced by hormonal changes with the potential to alter their physical performance [42]. Therefore, an analysis was performed adjusting for this variable and avoiding confounding factors. The results showed the influence of age (lower), DBP (higher value is associated with lower grip strength), height, ethnicity, depression, and IPAQ score. In clinical practice, dynamometric measurements of handgrip strength have been an important predictor of long-term mortality and disability [11,15,22]. However, in Colombia this measure is still rare, although the evidence found in the literature suggests that it would be a recommended tool for control programs at all levels of care.

As far as the association of depression with handgrip strength is concerned, the literature offers conflicting conclusions [42,43]. Our study found such an association even after adjusting for sex, recording a mean value of prehensile strength of 26.606 kg in people without depression, which decreases in people with mild (0.89 times *p* = 0.047) and moderate or severe depression (0.77 times *p* = 0.032). This finding is a call for the need to pay attention to mental clinical factors and their influence on physical parameters such as grip strength.

The regression model seeks to explain the values of handgrip strength in the population with HBP, highlighting the sociodemographic and clinical variables with more weight independent of sex: age (*p* = 0.043), depression (*p* = 0.025), and level of physical activity measured with IPAQ (*p* = 0.031). Different models have been proposed for healthy populations in terms of the variables that are considered final predictors; in the United States, a model was proposed that includes age, ethnicity, and income as sociodemographic variables in relation to grip strength [44], and in the Korean population, a model with BMI, exercise, nutritional status, house income, educational level, habits, and comorbidity [45]. Furthermore, the model proposed for the European population includes Old-age socio-economic and financial circumstances measured by wealth [46].

Finally, we identified some limitations in the study, such as the lack of examination of variables with potential to explain changes in prehensile strength values, among these markers of cardiometabolic risk such as increased fat, cholesterol, tri-glycerides, and atherogenic index values, all of which have already been established [42] as being associated with reduced muscle strength in a population without HBP. Other variables include dietary patterns, which have been associated with prehensile strength development [47], as well as sleep quality, which may also affect prehensile strength development [48]. The self-reporting of health variables such as smoking and alcohol consumption can induce information bias. Due to the cross-sectional nature of this study, the results should be interpreted with caution, as they correspond to estimates, and we cannot ascribe causality.

Further studies are needed on the associations between anthropometric measurements and follow-up studies to see changes over time in the same population with HBP. Likewise, the population without HBP could be evaluated to determine existing differences related to the pathology.

## 5. Conclusions

In a middle-aged HBP population, handgrip strength showed a strong direct association with the level of physical activity, and an inverse association with age and level of depression, independent of sex. People classified at the highest levels of depression (moderate/severe) showed the lowest strength values. The latter focuses attention on the need to address mental conditions and their influence on physical parameters, recognizing the role of handgrip strength not only in physical but also in mental health conditions. Likewise, mental health is positioned as a key factor in public health interventions focused on improving functionality, in this case of the upper limb based on handgrip strength.

## Figures and Tables

**Table 1:** Clinical and sociodemographic characteristics of participants.

Age	Variable	Category	Me */Mean **	IQR/SD	[95% CI]
Time since diagnosis (months)		7	6	6	8
SBP * (mm/Hg)		120 *	10	120	124.52
DBP ** (mm/hg)		78.17	±8.23	77.07	79.26
Weight * (kg)		71 *	20	70	74
Height ** (m)		1.6129	±0.089	1.6010	1.6249
BMI * (kg/m^2^)		27.63 *	5.90	26.87	28.59
Waist circumference * (cm)		93.50 *	18	92	96.52
METs * (Kcal)		495.00 *	1068	396	594
		*n*	%	CI 95%	
Sex	Woman	148	67.58	60.95	73.73
	Man	71	32.42	26.26	39.05
Educational attainment	None	5	2.28	0.75	5.25
	Primary	26	11.87	7.90	16.91
	Secondary	125	57.08	50.24	63.73
	Technical	44	20.09	14.99	26.02
	University	19	8.68	5.30	13.22
Occupation	Employed	79	36.07	29.71	42.82
	Retired	41	18.72	13.78	24.53
	Homemaker	62	28.31	22.45	34.77
	Permanent disability	2	0.91	0.11	3.26
	Unemployed	35	15.98	11.39	21.51
Marital status	Married	97	44.29	37.60	51.14
	In partnership	60	27.40	21.60	33.81
	Widowed	17	7.76	4.59	12.14
	Separated	11	5.02	2.53	8.81
	Single	34	15.53	11.00	21.01
HBP Medication	No	17	7.76	4.59	12.14
	Yes	202	92.24	87.86	95.41
Abdominal	No	32	14.61	10.21	19.9
obesity	Yes	187	85.38	80	89.78
Ethnicity	Black(a)/Mulatto(a)/Afro-Colombian/Afro	30	13.70	9.44	18.97
	Other	189	86.30	84.12	89.79
Hand Dominance	Right	200	91.32	86.78	94.70
	Left	13	5.94	3.20	9.94
	Ambidextrous	6	2.74	1.01	5.87
Smoking	No	202	92.24	87.86	95.41
	Yes	17	7.76	4.59	12.14
Alcohol	No	178	81.28	75.47	86.22
	Yes	41	18.72	13.78	24.53
Physical activity by IPAQ	Low	141	64.38	57.65	70.72
	Medium	57	26.03	20.35	32.37
	High	21	9.59	6.03	14.28
ZUNG	No depression	65	29.68	0.24	0.36
	Mild	106	48.40	42.3	55.2
	Moderate	47	21.46	16.4	27.6
	Severe	1	0.46	0.00	0.53

* Median Shapiro–Wilk test *p* < 0.05. CI: confidence interval. IQR: interquartile range. MCT: measures of central tendency. ** Mean (SD): standard deviation. SBP: systolic blood pressure. DBP: diastolic blood pressure. BMI: body mass index. MET: metabolic equivalent. HPB: high blood pressure medication. IPAQ: International Physical Activity Questionnaire.

**Table 2:** Handgrip strength values by sociodemographic and clinical variables.

Variable	Category	Handgrip Strength Median Value	*p* Value ¥	Effect Size (*r*)
Sex	Woman	21.58	0.000 ¥	0.73 *
	Man	38.33		
HBP medication	No	25.5	0.953 ¥	0.04
	Yes	24.66		
Smoking	No	24.58	0.017 ¥	0.48 **
	Yes	36.16		
Alcohol	No	24.16	0.016 ¥	0.29
	Yes	32		
Abdominal obesity	No	23	0.006 ¥	0.10
	Yes	25.33		
Educational attainment	None-primary	23	0.038 ¥¥	0.23
	Secondary	23.66		
	Technical	29.16		
	University	26.66		
Occupation	Employed	30	0.000 ¥¥	0.79 *
	Retired	23.5		
	Homemaker	21.33		
	Permanent disability	30		
	Unemployed	30.33		
Marital status	Married	23.66	0.061 ¥¥	0.36
	In partnership	28.91		
	Widowed	21.33		
	Separated	22.83		
	Single	22.83		
Ethnicity	Black(a)/Mulatto(a)/Afro-Colombian/Afro	31.75	0.019 ¥	0.31
	Other	23.83		
Hand Dominance	Right	24.66	0.906 ¥¥	0.02
	Left	29		
	Ambidextrous	25.83		
Physical activity by IPAQ	Low	23.5	0.000 ¥¥	0.96 *
	Medium	26.33		
	High	37.16		
Zung	No depression	25	0.041 ¥¥	0.61 *
	Mild	23.5		
	Moderate and severe	21.6		

¥ Non-parametric test in order to compare two independent groups: Wilcoxon rank-sum (Mann–Whitney) *p* < 0.05 describes significant differences. ¥¥ Non parametric test in order to compare two or more independent groups: Kruskal–Wallis *p* < 0.05 describes significant differences. * Large effect size (r equal to or greater than 0.5); ** medium effect size (equal to or greater than 0.3).

**Table 3:** Association between handgrip strength and each sociodemographic and clinical variable (bivariate analysis).

FDER		β Value	*p* > z	[95% CI]	
Sex	Man	1.76	0.000 *	1.63	1.91
	β0 (Woman)	21.61	0.000	0.000	22.60
Age β0 (34 years)	--	0.98 34.55	0.001 * 0.000	0.98 29.38	0.99 40.63
Marital status	In partnership	1.08	0.220	0.95	1.22
	Widowed	0.802	0.029 *	0.65	0.97
	Separated	0.81	0.085 *	0.63	0.89
	Single	1.08	0.31	0.93	12.54
	β0 (Married)	26.73	0.00	24.77	28.85
Educational attainment	Secondary	1.168	0.048 *	1.05	1.36
	Technical	1.30	0.004 *	1.08	1.55
	University	1.22	0.072 *	1.18	1.53
	β0 (None or primary)	23	0.000	20.00	26.32
Occupation	Retired	0.83	0.007 *	0.73	0.95
	Homemaker	0.67	0.000 *	0.60	0.76
	Permanent disability	0.97	0.917	0.59	1.58
	Unemployed	0.99	0.913	0.86	1.13
	β0 (Employed)	30.78	0.000	28.52	33.23
Ethnicity	Other β0 (Afro-Colombiano)	0.84 31.25	0.026 * 0.000	0.72 27.14	0.97 3.99
Hand dominance	Left	1.071	0.541	0.857	1.339
	Ambidextrous	1.056	0.737	0.765	1.458
	β0 (Right)	26.837	0.000	25.400	28.35
IPAQ rating	Medium	1.174	0.007 *	1.045	1.320
	High	1.436	0.000 *	1.207	1.709
	β0 (Low)	24.824	0.000	23.317	26.427
METs(Kcal) β0 adjusted to median 495	--	1.000 26.6	0.055 0.000	0.999 24.817	1.000 27.862
Depression	Mild	0.895	0.047 *	0.880	0.920
	Moderate or severe	0.776	0.032 *	0.635	0.789
	β0 (without depression)	26.606	0.000	24.158	29.301
HPB medication	Yes β0 No	1.01 26.64	0.886 0.000	0.833 22.056	1.234 32.169
Smoking	Yes	1.230	0.037 *	1.012	1.496
	β0 No	26.519	0.000	25.113	28.002
Alcohol	Yes	1.212	0.004 *	1.064	1.381
	β0 No	25.961	0.000	24.536	27.467
Time since diagnosis (months) β0 (6 months)	--	0.996 27.194	0.436 0.000	0.988 25.716	1.004 28.756
SBP (mmHg) β0 (SBP 120)	--	0.996 27.424	0.153 0.000	0.992 25.902	1.001 29.035
DBP (mmHg) β0 (DBP 80)	--	0.991 26.537	0.012 * 0.000	0.985 25.170	0.998 27.978
Weight β0 (75 kg)	--	1.010 26.789	0.000 * 0.000	1.007 25.539	1.013 28.100
Height β0 (161 cm)	--	1.024 26.113	0.000 * 0.000	1.020 25.059	1.029 27.124
BMI β0 (28)		1.004 26.907	0.400 0.000	0.993 25.520	1.015 28.370
Waist circumference β0 (80 cm)		1.006 24.48	0.102 0.000	1.000 22.61	1.009 26.51

β0: handgrip strength in reference category. * Significant association (*p* < 0.05). CI 95%: confidence interval of 95%. SBP: systolic blood pressure. DBP: diastolic blood pressure. BMI: body mass index. MET: metabolic equivalent. IPAQ: International Physical Activity Questionnaire.

**Table 4:** Association between handgrip strength and the clinical and sociodemographic characteristics of participants (multiple model).

HANDGRIP STRENGTH		Exp(β1)	*p* > z	[95% CI]	
Age		0.967	0.043 *	0.954	0.992
DBP		0.996	0.094	0.991	1.000
Weight		1.001	0.277	0.998	1.004
Height		1.007	0.013	1.005	1.012
Occupation	Retired	0.917	0.124	0.823	1.023
	Homemaker	0.986	0.807	0.884	1.099
	Permanent disability	1.017	0.926	0.701	1.476
	Unemployed	0.990	0.862	0.884	1.102
Marital status	In partnership	1.01	0.778	0.926	1.106
	Widowed	1.071	0.358	0.924	1.241
	Separated	1.044	0.612	0.881	1.237
	Single	1.117	0.041 *	1.004	1.243
IPAQ	Medium	1.175	0.045 *	1.163	1.179
	High	1.240	0.031 *	1.151	1.294
Depression (Zung Scale)	Mild	0.899	0.045 *	0.815	0.901
	Moderate and severe	0.797	0.025 *	0.773	0.799
β0		23.943	0.000	20.006	28.654

SBP: systolic blood pressure. DBP: diastolic blood pressure. BMI: body mass index. MET: metabolic equivalent. IPAQ: International Physical Activity Questionnaire. The analysis employed age centered around 34 years (its minimum value), DBP around 80 mmHg, weight around its average (75 kg), and height around 161 cm. Categories of reference: occupation (employed), type of occupation (unqualified manual labor), marital status (married), ethnicity (Black/Afro Colombian), IPAQ (low) and depression (without depression). * Significant differences.

## Data Availability

The data shown in this study are available upon request from the corresponding author. The data are not available to the public, since taking into account the sensitive nature of all the questions asked in this study, all participants were guaranteed that the data obtained would be confidential and would not be shared.
